# Occult neck metastases risk factors and the role of elective neck dissection in cT3-4N0 adenoid cystic carcinoma of the parotid gland

**DOI:** 10.3389/fonc.2022.935110

**Published:** 2022-09-26

**Authors:** Junhui Yuan, Fan Meng, Chunmiao Xu, Wenlu Li, Shuang Wu, Hailiang Li

**Affiliations:** ^1^ Department of Radiology, The Affiliated Cancer Hospital of Zhengzhou University and Henan Cancer Hospital, Zhengzhou, China; ^2^ Department of Stomatology, The Affiliated First Hospital of Zhengzhou University, Zhengzhou University, Zhengzhou, China

**Keywords:** adenoid cystic carcinoma, parotid gland malignancy, elective neck dissection, observation, survival

## Abstract

**Objectives:**

To determine the predictor for occult neck metastases and the role of elective neck dissection (END) in cT3-4N0 parotid adenoid cystic carcinoma (ACC).

**Methods:**

Patients with surgically treated parotid ACC were retrospectively enrolled. Predictors of occult neck metastases and the effect of END on disease specific survival (DSS), overall survival (OS), locoregional control survival (LRC), and distant metastasis free survival (DMS) were analyzed.

**Results:**

Occult neck metastases occurred in 35 (19.7%) of the 178 patients undergoing an END. The tumor stage [p=0.011, 4.215 (1.387–10.435)] and intra-parotid lymph node metastasis [p=0.032, 3.671 (1.693–8.775)] were related to the possibility of occult neck metastases independently. The END group had better 10-year LRC than the observation group (56% vs. 43%, p=0.002) and also better 10-year DMS than the observation group (43% vs. 32%, p<0.001). The two groups had similar 10-year DSS (40% vs. 33%, p=0.230) and OS (31% vs. 23%, p=0.094) rates. Furthermore, the Cox model confirmed that END was independently associated with better LRC rate [p=0.022, 2.576 (1.338–6.476)] and better DMS [p=0.011, 2.343 (1.274–7.462)].

**Conclusions:**

Occult neck metastases in cT3-4N0 parotid ACC was not common. A T4 tumor with intra-parotid lymph node metastasis had the highest possibility of occult neck metastases. END had no effect on DSS or OS but significantly decreased the risk of locoregional recurrence and distant metastasis.

## Introduction

Adenoid cystic carcinoma (ACC) is one of the most common parotid gland malignancies in the head and neck (1). It is characterized by slow growth, perineural invasion (PNI), and distant metastasis (2). Radical excision of the primary tumor is the preferred treatment, but owing to the wide range of the neck lymph node metastasis rate, the necessity for elective neck dissection (END) still remains controversial (3).

A review showed that the metastasis rate ranges from 0% to 43.7% based on 2,450 ENDs, and it concluded that ENDs could not provide any benefit in overall survival (OS) or disease specific survival (DSS) but could only be associated with a prolonged regional recurrence-free period (4). However, some researchers have reported that the overall neck lymph node metastasis rate is about 10%, and it varies with the primary site and tumor stage. cT3-4 ACC located in the oral cavity or oropharynx has the highest probability of lymph node metastasis. END could extend the OS (5–7). This viewpoint is confirmed by current evidence (8). Lymph node involvement is a risk factor for subsequent distant metastasis, and distant metastasis is the main cause of failure. Advanced tumor stage, the tongue-mouth floor complex as the primary site, and lymphovascular invasion (LVI) have been demonstrated to increase the risk of nodal metastasis (9–11). But it must be kept in mind that the occult neck metastases rate in cT3-4 ACC is still not very high. Routine END is related to over-treatment in many patients (4, 5, 10). Therefore, it is important to detect patients with occult neck metastases at initial treatment. Moreover, these factors are not validated in parotid ACC, and there are other variables not studied, including intra-parotid lymph node (IPN).

Therefore, the current study aimed to assess the predictor for occult neck metastases and the role of END in cT3-4N0 parotid ACC using relatively large sample size.

## Patients and methods

### Ethical considerations

The institutional research committee approved this study, and all participants signed an informed consent agreement. All procedures performed in this study involving human participants were in accordance with the ethical standards of the institutional and national research committee. The Helsinki Declaration (1964) and its later amendments or comparable ethical standards were followed.

### Patient selection

Medical records of patients with surgically treated parotid ACC were retrospectively reviewed between January 2000 and January 2022. The inclusion criteria of the patients included patients with primary ACC staged as cT3-4N0 according to the 8^th^ American Joint Committee on Cancer classification system with available follow-up data. Patients with a history of other malignancies were excluded. Information on demography, pathology, treatment, and follow-up was extracted and analyzed.

### Treatment principle

Systematic examinations were routinely performed for every patient, including ultrasound, computed tomography, magnetic resonance imaging, and/or positron emission tomography-computed tomography scan. Total parotidectomy with or without facial nerve preservation was performed for all patients. END was selectively performed based on the surgeon’s preference, the patient’s status, and imaging analysis. Post-operative radiotherapy is usually advised in our cancer center when there is a presence of parotid ACC. After therapy, the patients were required to be examined every 3 months during the 1st year, every 6 months during the 2nd year, and once per year after the 2nd year by outpatient clinic visits, telephone calls, emails, or WeChat messages (12).

### Statistical analysis

The association between occult neck metastases and clinicopathologic variables was evaluated *via* univariate analysis (the Chi-square test) and multivariate analysis (the logistic regression model). The endpoints were locoregional control (LRC), distant metastasis free survival (DMS), OS, and DSS. The LRC was calculated from the date of surgery to the date of locoregional recurrence or the last follow-up, and the DMS was calculated from the date of surgery to the date of distant metastasis or the last follow-up. The OS was calculated from the date of surgery to the date of death or the last follow-up. The DSS was calculated from the date of surgery to the date of death caused by the disease or the last follow-up. The Kaplan–Meier method was used to calculate the LRC, DSS, and OS rates. The Cox proportional hazards regression model was used for multivariate analysis. All statistical analyses were performed using IBM SPSS Statistics for Windows, version 20.0 (IBM Corp., Armonk, N.Y., USA), and a p<0.05 was considered significant.

## Results

Overall, 178 patients underwent an END, and 76 patients did not. In total, there were 100 males and 154 females, with a mean age of 48.6 ± 13.8 years. Primary tumors were located in the superficial lobe in 176 patients and in the deep lobe in 78 patients. Tumor stages were distributed as T3 in 157 patients and T4 in 97 patients. A clean margin was achieved in 235 patients. At initial treatment, distant metastasis occurred in 14 patients. PNI and LVI were noted in 84 and 57 patients, respectively. IPN metastasis occurred in 18 patients. All patients received radiotherapy for their primary sites, and neck radiation therapy was performed on 85 patients.

### Incidence of occult neck metastases

**Figure 1:**
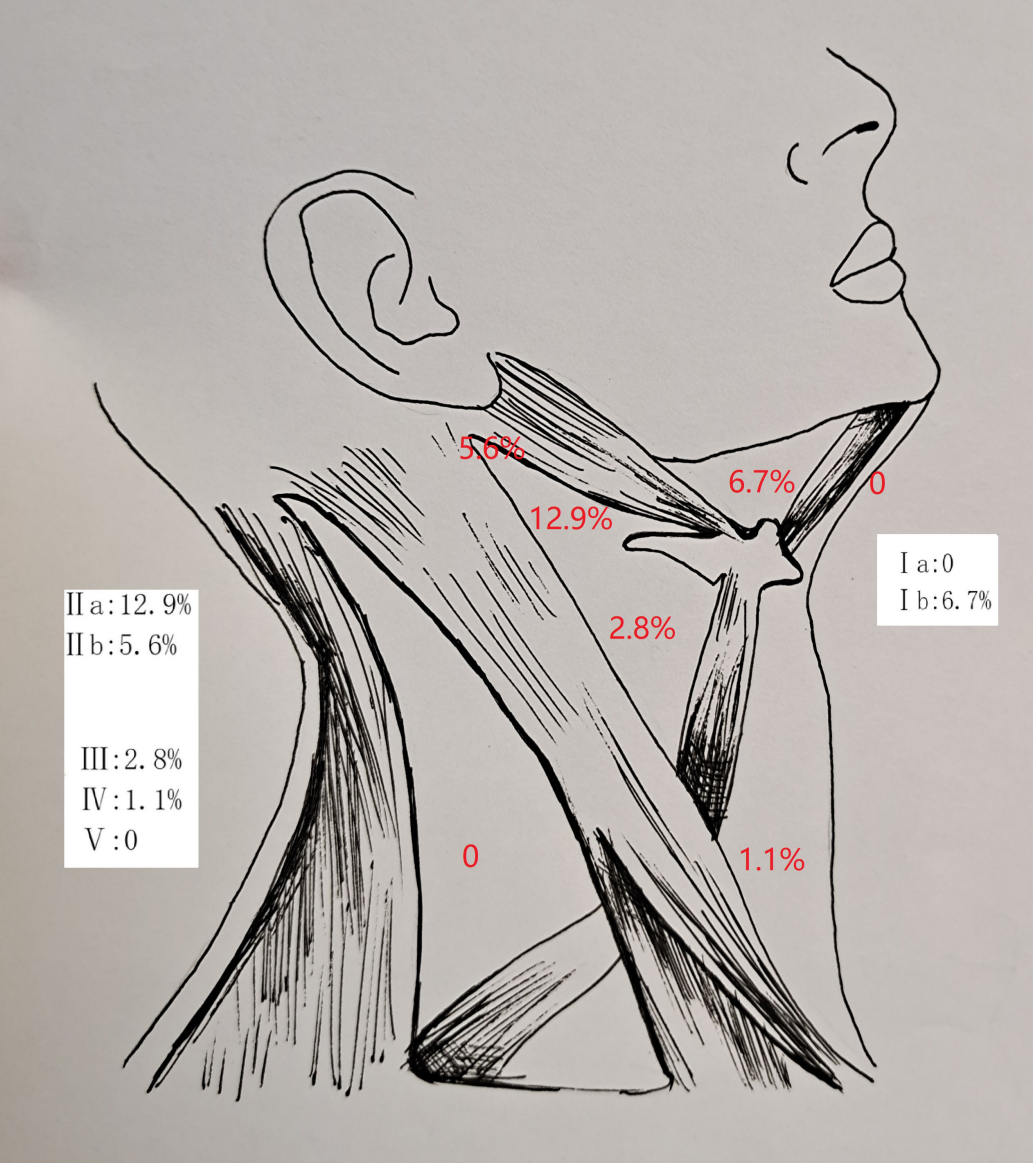
Lymph node metastasis pattern in cT3-4N0 parotid adenoid cystic carcinoma.

In the 178 patients undergoing an END, an occult neck metastases occurred in 35 patients, with a rate of 19.7%. The mean number of positive neck lymph nodes was 2.5 ± 1.5. There was no contralateral neck metastasis. The involvement of levels IIa, Ib, IIb, III, and IV was noted in 23, 12, 10, 5, and 2 patients, respectively. There was no metastasis at level Ia or level V (**Figure 1**).

### Risk factors of occult neck metastases

ACC in the deep lobe had an occult neck metastases rate of 28.3%. It was significantly higher than that of ACC in the superficial lobe (p = 0.033). Occult neck metastases occurred in 27.8% of T4 tumors and in 14.2% of T3 tumors. The difference was significant (p = 0.025). In 10 patients with IPN metastasis, five patients had occult neck metastases, but in 168 patients without IPN metastasis, only 30 patients had occult neck metastases. The difference was significant (p = 0.027). No significant association was noted between occult neck metastases and other clinicopathologic variables (**Table 1**).

**Table 1:** Univariate analysis of risk factor of occult metastasis.

Variable	Occult metastasis		p
	Yes (n=35)	No (n=143)	
Age			
<50	17	80	
≥50	18	63	0.432
Sex			
Male	15	65	
Female	20	78	0.782
Tumor location			
Superficial	17	97	
Deep	18	46	0.033
Tumor stage			
T3	15	91	
T4	20	52	0.025
Distant metastasis			
Yes	3	6	
No	32	137	0.382
Perineural invasion			
Yes	14	53	
No	21	90	0.748
Lymphovascular invasion			
Yes	10	27	
No	25	116	0.205
Intraparotid lymph node metastasis			
Yes	5	5	
No	30	138	0.027

In further multivariate analysis, tumor stage [p = 0.011, 4.215 (1.387–10.435)] and IPN metastasis [p = 0.032, 3.671 (1.693–8.775)] were independently related to the possibility of occult neck metastases (**Table 2**).

**Table 2:** Multivariate analysis of risk factor of occult metastasis.

Variable	OR [95%CI]	p
Tumor location	3.667 [0.674-9.227]	0.243
Tumor stage	4.215 [1.387-10.435]	0.011
Intraparotid lymph node metastasis	3.671 [1.693-8.775]	0.032

### Effects of END on survival

**Figure 2:**
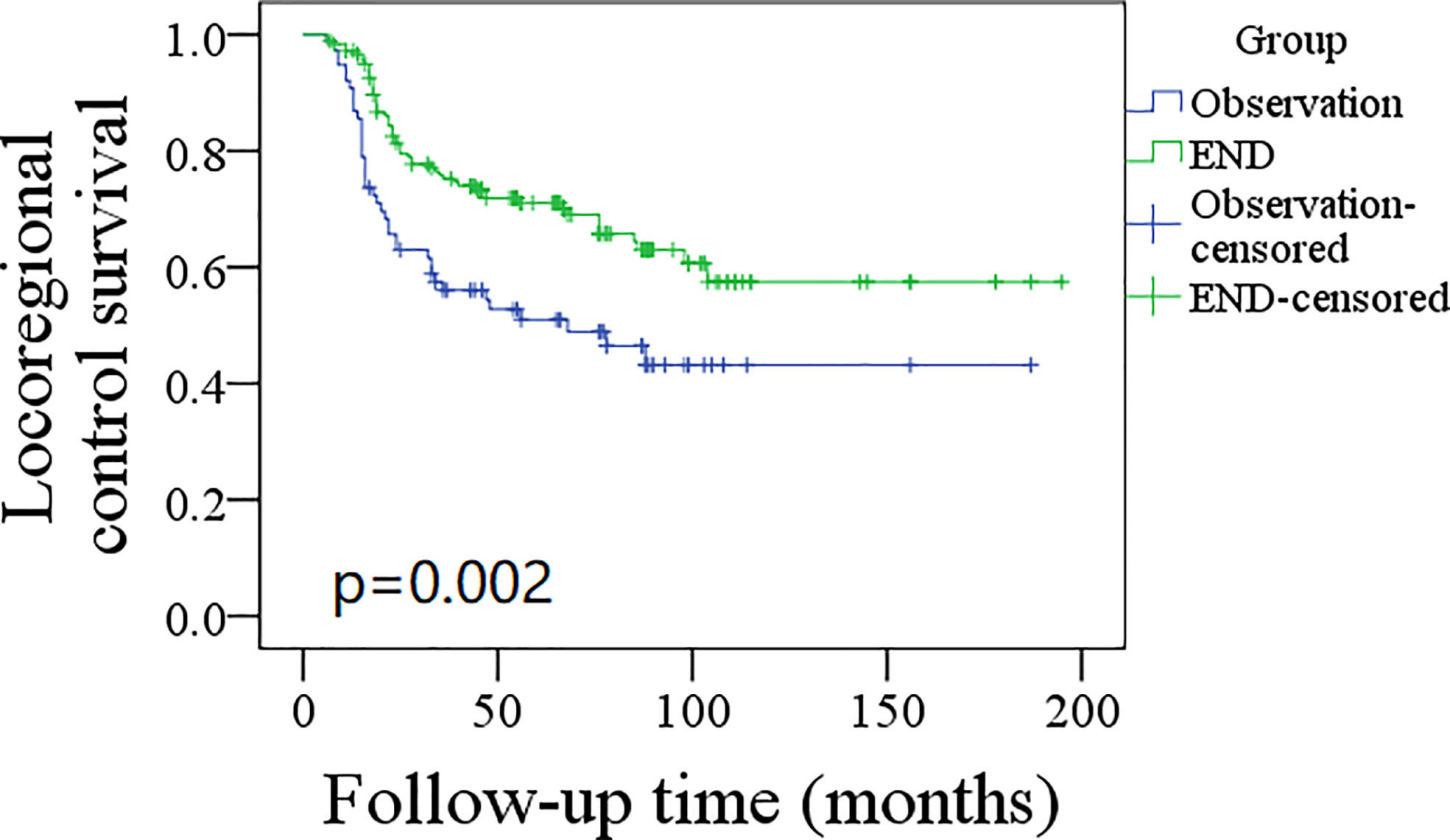
Comparison of locoregional control survival between elective neck dissection (END) and observation groups (p = 0.002).

**Figure 3:**
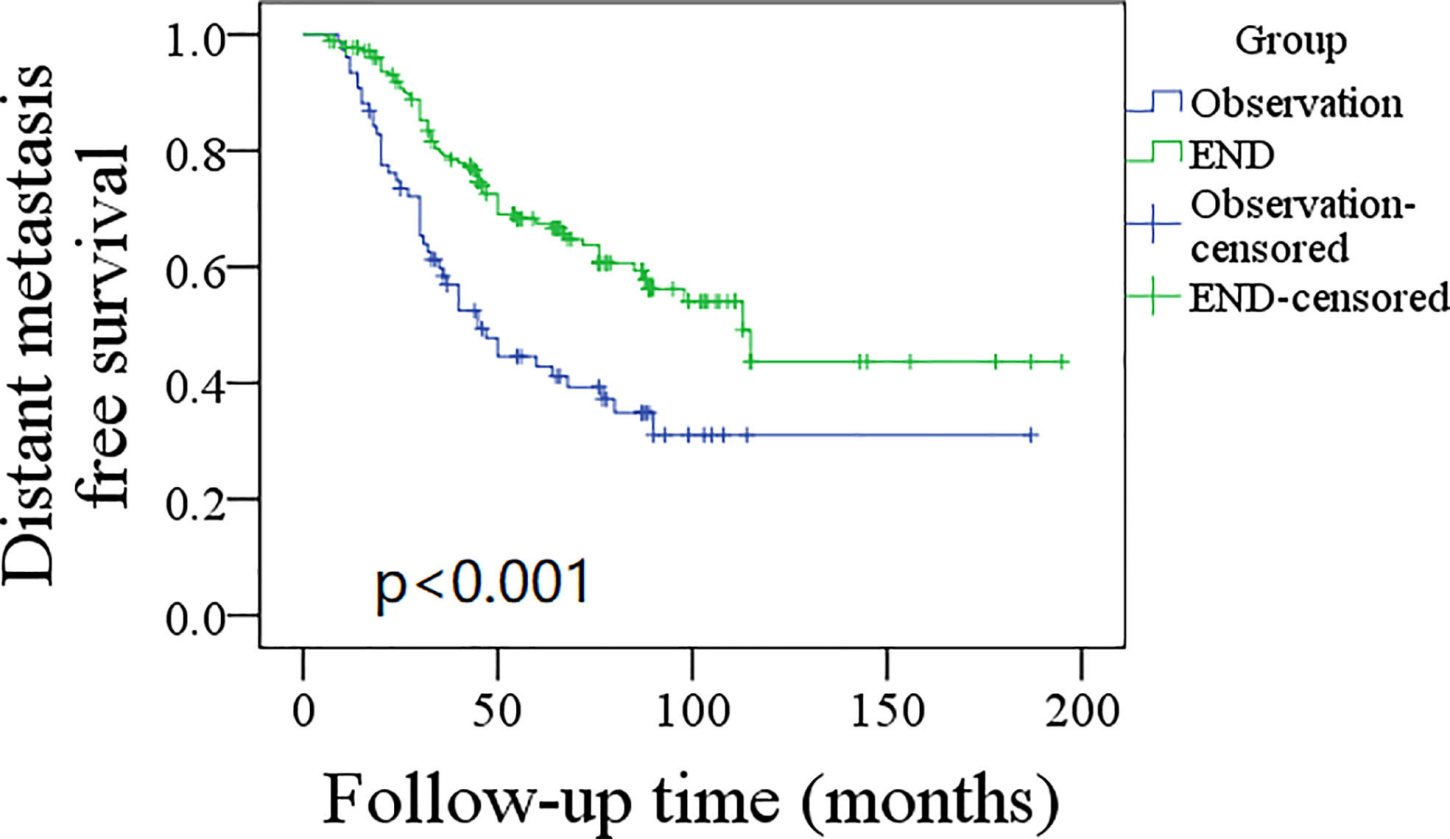
Comparison of distant metastasis free survival between elective neck dissection (END) and observation groups (p < 0.001).

**Figure 4:**
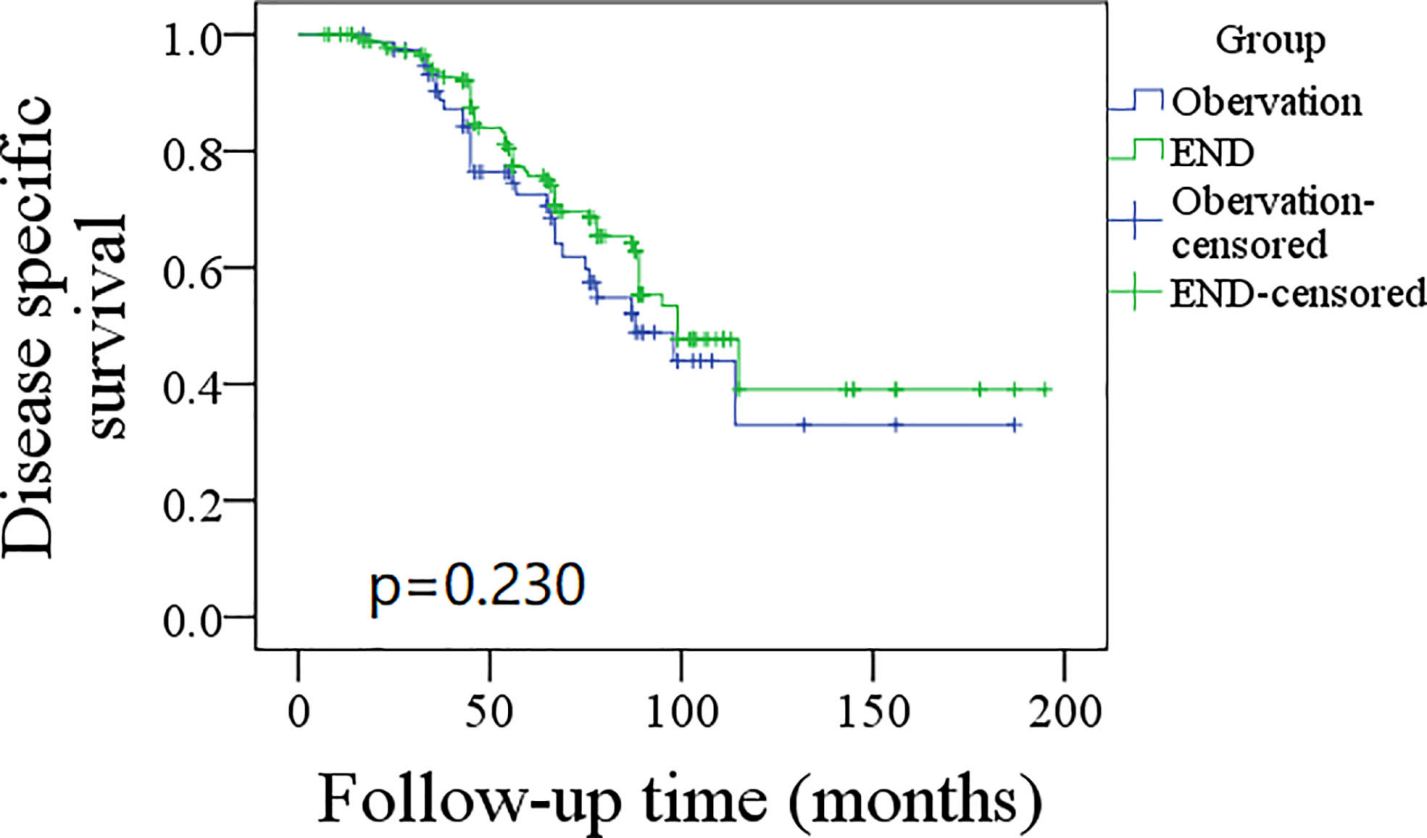
Comparison of disease specific survival between elective neck dissection (END) and observation groups (p = 0.230).

**Figure 5:**
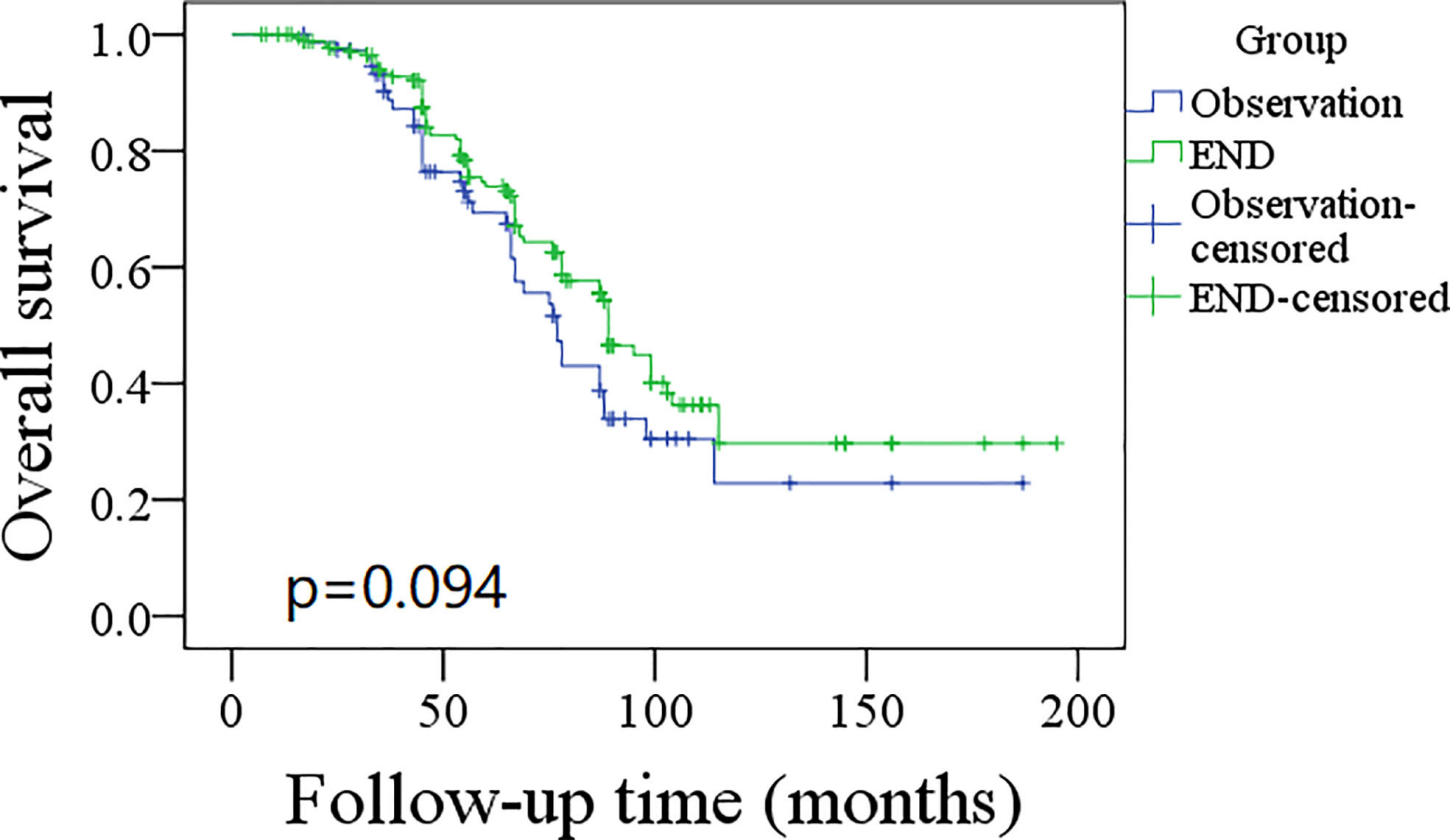
Comparison of overall survival between elective neck dissection (END) and observation groups (p = 0.094).

A total of 76 patients received an observation policy for neck management. During the follow-up with a mean time of 67.1 ± 33.1 months, 37 patients had locoregional recurrence, and 46 patients developed distant metastasis. Of these, 39 patients died, of whom 30 died of the disease. In the END group, 57 patients had locoregional recurrence, 65 patients developed distant metastasis, and 74 patients died, of whom 60 died of the disease. The END group had a better 10-year LRC than the observation group (56% vs. 43%, p = 0.002, **Figure 2**) and a better 10-year DMS than the observation group (43% vs. 32%, p<0.001, **Figure 3**). The two groups had similar 10-year DSS (40% vs. 33%, p = 0.230, **Figure 4**) and OS (31% vs. 23%, p = 0.094, **Figure 5**) rates. In a further Cox model, END was independently associated with a better LRC rate [p = 0.022, 2.576 (1.338–6.476)] and better DMS [p = 0.011, 2.343 (1.274–7.462)] (**Tables 3** , **4**).

**Table 3:** Prognostic factors of locoregional control survival in patients with parotid adenoid cystic carcinoma.

Variable	Univariate	Cox model	
		RR [95%CI]	p
Age	0.732		
Sex	0.631		
Tumor location	0.422		
Tumor stage	0.012	4.278 [1.674-10.337]	0.013
Perineural invasion	<0.001	3.984 [0.746-9.658]	0.111
Lymphovascular invasion	0.178		
Intraparotid lymph node metastasis	0.032	3.224 [1.925-8.115]	0.005
Neck management	0.002	2.576 [1.338-6.476]	0.022
Neck radiation therapy	0.373		

**Table 4:** Prognostic factors of distant metastasis free survival in patients with parotid adenoid cystic carcinoma.

Variable	Univariate	Cox model	
		RR [95%CI]	p
Age	0.492		
Sex	0.366		
Tumor location	0.243		
Tumor stage	0.023	3.167 [1.777-10.263]	0.006
Perineural invasion	0.336		
Lymphovascular invasion	0.038	2.683 [1.054-5.733]	0.032
Intraparotid lymph node metastasis	0.032	3.465 [0.917-10.476]	0.258
Neck management	<0.001	2.343 [1.274-7.462]	0.011
Neck radiation therapy	0.551		

## Discussion

To our knowledge, this was one of the largest series regarding cT3-4N0 parotid ACC. The most important finding in the current study was that the occult neck metastases in cT3-4N0 parotid ACC was not common. A T4 tumor with IPN metastasis had the highest possibility of occult neck metastases. The current findings demonstrate that END decreased the risk of locoregional recurrence and distant metastasis, but the two groups had similar DSS and DS.

Occult neck metastases in the head and neck ACC were extensively analyzed. Amit et al. (13) found that oral ACC had a nodal metastasis rate of 22% based on 226 cases. It was significantly higher than 16% in those in the paranasal sinuses and 12% in those in major glands. Min et al. (11) also described the occult neck metastases rates of ACC in the tongue-mouth floor complex and other sites as 17.2% and 8.2%, respectively. The difference was significant. These findings highlighted that the occult neck metastases rate in major glands might be low. Megwalu et al. (10) reported that the overall nodal metastasis rate of ACC in major glands was 17.3%, and tumor stage but not the primary site was associated with nodal metastasis. However, because the study did provide information regarding the ratio of cN+ to cN0, the incidence of occult neck metastases could not be calculated. In another study by Qian et al. (14) using the National Cancer Center Database, the occult neck metastases rate was only 9.0% in major gland ACC, but the metastasis rate in parotid ACC was not given. A review by an international head and neck scientific group showed the prevalence of positive nodes from ACC was 14.5% for the parotid gland, 22.5% for the submandibular gland, and 24.7% for the sublingual gland, and the only significant factor contributing to nodal metastasis was tumor stage (15). Considering the intrinsic nature of ACC neoplasms, it arose from a non-capsulated organ such as the parotid gland; thus, its infiltrative growth was not hindered, letting ACC invade adjacent tissue without well-defined borders (16). More complex and different metastasis patterns could be expected. This study focused on predictors of occult neck metastases in cT3-4N0 parotid ACC and might be the first to note the significance of IPN metastasis.

The pooled prevalence of IPN metastasis in primary parotid malignancies was 24.1%, and it was most likely to occur in those high-grade cancers excluding ACC (17). A series of reports by Fang et al. (18–20) also confirmed the association between IPN metastasis and cervical nodal metastasis in parotid cancers, but the cases of ACC were very limited. The current study found that the overall occult neck metastases rate was only 19.7%. Generally, END was suggested if the probability of occult cervical metastasis was greater than 15%–20% in head and neck squamous cell carcinoma (21). Advanced tumor stage was described to be associated with increased nodal metastasis risk and END was suggested in cT3-4 parotid malignancies (10, 11, 13, 14). Based on the current findings, routine END in cT3-4N0 parotid ACC caused some degree of overtreatment. IPN had a high specificity (96.5%) in predicting nodal metastasis; a frozen section of IPN would help in neck decision making and decrease the unnecessary surgical burden.

The cervical nodal metastasis pattern was another important issue; it formulated the dissection range. In 82 positive neck dissection samples by Zhang et al. (5), levels I and II metastasis were most common, occurring in 52 and 32 cases, respectively. Level IV metastasis was rare. A review by Luksic et al. (4) concluded that these metastases were most often solitary unilateral metastatic deposits (pN1 disease), located in most cases within levels I–III. Amit et al. (13) revealed ipsilateral nodal metastases in 44 patients (16%), consisting of 33 in levels I to III and 11 in levels IV to V (all on the ipsilateral side). Our result was consistent with these findings; it supported that END should at least include levels I–III to clean the metastasis foci, and the possibility of level IV to V metastasis could not be ignored.

The survival benefit, added by END to the head and neck ACC, remains controversial (22). Xiao et al. (7) analyzed the role of END in 2,807 patients with head and neck ACC. They found that patients with advanced T3 to T4 ACC of the major salivary gland demonstrated extended OS associated with END in univariate analysis and with END combined with adjuvant radiotherapy in multivariate analysis. However, the two groups showed apparent differences in multiple clinicopathologic factors, which caused unreliability in comprehending the finding. More researchers reported conflicting results. Amit et al. (23) enrolled 457 patients with cN0 head and neck ACC, of whom 226 cases received END, and 231 did not. The two groups had comparable 5-year OS (72% vs. 79%) and 5-year DSS (74% vs. 81%); however, the two groups had significant differences regarding baseline variables of the primary site and adjuvant chemotherapy, which both affected the survival outcome. Lee et al. (24) retrospectively evaluated the outcomes of 61 patients with salivary gland ACC; 26 patients received END, during their follow-up, regional recurrence only occurred in patients without END at initial treatment, but there were no significant differences in distant metastasis or OS between the two groups. However, the sample size of this study was very small, and T1 to T4 tumors were mixed together, nodal metastasis in early-stage ACC was uncommon, and END was relatively more required in advanced-stage tumors than in early-stage disease. A recent meta-review also described that compared to observation, END did not provide any benefit in OS irrespective of tumor stage (25). However, as the authors pointed out, these results must be taken with caution due to the high heterogeneity of the data and the lack of stratification among END and no END subgroup based on the T stage or histological subtype with respect to survival, which could influence the obtained results. The current study overcame these shortcomings and confirmed END was not associated with improved OS or DSS in cT3-4N0 parotid ACC, but there was an interesting finding that END provided better LRC and DMS. Removing positive lymph nodes and blocking the pathway of lymph node metastasis in time could decrease the risk of regional recurrence (26). Conversely, evidence of lymphatic spread increased the rate of distant metastases (4, 13), and END improved DMS. The finding was rarely reported; only Amit et al. (23) previously described that END and observation groups had similar distant metastasis rates. The difference could be explained by the fact that the latter analyzed ACC in all subsites in the head and neck together; there was a high heterogeneity.

The current study has a few limitations. First, the retrospective study had inherent bias. Second, the molecular mechanism of regional recurrence and distant metastasis was not analyzed.

In summary, occult neck metastases in cT3-4N0 parotid ACC were not common. A T4 tumor with IPN metastasis had the highest possibility of occult neck metastases. It has been observed that END had no effect on DSS or OS but significantly decreased the risk of locoregional recurrence and distant metastasis. Every patient should be approached individually and the decision on the performance of END in cT3-4N0 parotid ACC should be made after multidisciplinary discussion until new evidence/data on the topic occurs, and the dissected neck level should be limited to II–IV.

## Data availability statement

The original contributions presented in the study are included in the article/Supplementary Material. Further inquiries can be directed to the corresponding author.

## Ethics statement

This study was reviewed and approved by Henan Cancer Hospital (HNCY21778). Written informed consent was obtained from all participants for their participation in this study.

## Author contributions

The authors made all the contribution of study design, manuscript writing, studies selecting, data analysis, study quality evaluating, and manuscript revising. The final manuscript was read and approved.

## Funding

The study was supported by Special fund for clinical research of Wu Jieping MedicalFoundation (320.6750.2020-08-8).

## Conflict of interest

The authors declare that the research was conducted in the absence of any commercial or financial relationships that could be construed as a potential conflict of interest.

## Publisher’s note

All claims expressed in this article are solely those of the authors and do not necessarily represent those of their affiliated organizations, or those of the publisher, the editors and the reviewers. Any product that may be evaluated in this article, or claim that may be made by its manufacturer, is not guaranteed or endorsed by the publisher.
